# An ultra-wideband orthogonal-beam directional graphene-based antenna for THz wireless systems

**DOI:** 10.1038/s41598-022-26398-2

**Published:** 2022-12-22

**Authors:** Sasmita Dash, Constantinos Psomas, Amalendu Patnaik, Ioannis Krikidis

**Affiliations:** 1 Department of Electrical and Computer Engineering, University of Cyprus, Nicosia, Cyprus; 2 Department of Electronics and Communication Engineering, Indian Institute of Technology Roorkee, Roorkee, India

**Keywords:** Electrical and electronic engineering, Electronic and spintronic devices

## Abstract

In terahertz (THz) wireless systems, graphene shows a tremendous promise for the implementation of miniaturized and reconfigurable antennas due to its unique tunable property. This paper presents a wideband beam reconfigurable directional antenna for THz wireless communication systems. The antenna design concept is based on the Yagi-Uda antenna working principle. The proposed antenna consists of a centre-fed graphene-based driven dipole and three graphene-based parasitic elements on either side of the driven element. These graphene-based parasitic elements either act as directors or reflectors by controlling the surface conductivity of these elements. The surface conductivity of the elements is adjusted individually by applying a bias voltage via the chemical potential of the graphene. The main beam direction of the antenna can be reconfigured by controlling the chemical potentials of the graphene-based parasitic elements. Specifically, the proposed graphene THz antenna reconfigures the main beam into four orthogonal directions (0°, 90°, 180° and 270°) at an operational frequency 1.25 THz. The antenna achieves a directional symmetrical radiation pattern with 14 dBi gain and a front-to-back ratio of 15.5 dB. Furthermore, the proposed graphene THz antenna provides a stable reflection coefficient in four reconfigurable cases and an ultra-wideband of 10-dB impedance bandwidth of 120%. Therefore, this novel design technique of graphene-based ultra-wideband high gain beam reconfigurable THz antenna is promising for THz wireless systems.

## Introduction

The use of the terahertz (THz) band, i.e., frequency ranging from 0.1 to 10 THz, has a great interest in wireless communications due to large bandwidth and high data rates^1^. The THz wireless communications have the advantage of more bandwidth, more directionality, and higher data rate compared to the radio frequency and microwave bands. However, the THz band has a high propagation loss, including molecular absorption loss^2^. Given the high propagation losses in THz wireless communications, the design of a THz antenna plays an important role since THz antennas of high directionality at both transmitter and receiver can compensate for propagation loss. Recently, several antenna designs at THz frequency have been reported in the literature^3–15^. However, conventional metal is no longer suitable due to a decrease in conductivity and skin depth in the THz band^16^. On the other hand, the nanomaterial graphene is a promising candidate for the implementation of THz miniaturized antennas due to the propagation of surface plasmon polaritons (SPP) waves in the THz regime^16^. Specifically, graphene has gained momentum for reconfigurable THz antennas due to its inherent tunable high conductivity^3^ . Recently, reconfigurable antennas based on graphene material at THz band have been widely explored^3–7,17^.

Yagi-Uda antenna is a well-known antenna design for the implementation of pattern reconfigurable antennas^18–20^. In the classical Yagi-Uda antennas, the reconfiguration is achieved by employing the switches on the parasitic elements. However, this conventional switch mechanism is not suitable in the THz band. The insertion of switches in THz implementation can increase the complexity of the antenna structure. By exploiting the tunable dynamic conductivity of graphene at THz, few radiation pattern reconfigurable antennas have been designed^21–23^. These antennas have dynamic radiation patterns that can be reconfigured, but the adjustable range for the radiation beam is limited.

The Yagi-Uda antenna concept has been used to design graphene-based reconfigurable pattern THz antennas^21,24^. However, the design process of a graphene-based Yagi-Uda THz antenna is more complicated. In^21^, the antenna only reconfigures the radiation beam from $-\,42^{\circ }$ to $+\,42^{\circ }$, and in^24^, the authors designed a complicated structure for a reconfigurable four-beam antenna using the Yagi-Uda antenna concept with metal as radiator and graphene as switches for reconfiguration. A design for a switched beam graphene THz antenna using Yagi-Uda concept has been provided in^25^. In this work, each parasitic element consists of three different graphene strips and are separated by a small distance of 500 nm, which increases the complexity in the antenna structure. The conductivity of graphene strips of each parasitic element adjusted to make the overall length of the parasitic element larger/smaller than the length of the excited dipole, and hence act as a reflector/director of the antenna structure. The bandwidth information of these beam reconfigurable antennas is not available in most of the literature. However, the 10-dB impedance bandwidth of the four-beam reconfigurable antenna in^24^ and^25^ are 10% and 42%, respectively. In^26^, multi-beam reconfigurable antenna based on graphene at 1.243 THz achieved 10-dB impedance bandwidth of 10.5%, and the gain of 6.5 dB with a 12.1 dB front to-back ratio. A beam-scanning seven-element quasi-Yagi-Uda antenna based on hybrid metal-graphene materials is deflected from $-7\,0^{\circ }$ to $+\,70^{\circ }$ by controlling the bias voltage of graphene in^27^. An array of graphene-based Yagi-Uda antennas is reported in^28^, which consists of four identical Yagi-Uda antennas, reconfigures the radiation beam from $-\,75^{\circ }$ to $+\,75^{\circ }$ with directivity of 9.78 dBi at the 2.5 THz resonant frequency with 12.38% operating wide bandwidth.

In comparison to previous works, in this report, we present a novel and simple way to design a graphene Yagi-Uda antenna with wideband and beam reconfiguration capability in the THz band. In this work, each parasitic element is a single graphene strip of dynamic conductivity, which makes its implementation simpler than previous works. Moreover, the proposed antenna provides wide bandwidth of 120% and high directivity of 14 dBi compared to previous works. The antenna is capable of reconfiguring its radiation pattern, covering a $360^{\circ }$ angle. The main beam direction of the graphene THz antenna is achieved by controlling the chemical potentials of the parasitic elements. In particular, the main contributions of the paper are summarized as follows.

- We propose a simple structured wideband beam reconfigurable THz antenna using novel graphene nanomaterial. The antenna design concept is based on the Yagi-Uda antenna principle. The antenna consists of graphene-based driven dipole and parasitic elements.
- We design a graphene-based Yagi-Uda reconfigurable antenna by using the electromagnetic (EM) simulator CST Studio Suite and provide the simulation technique for the EM modelling of graphene elements and the beam reconfiguration for the proposed antenna structure.
- We investigate the performance of the proposed antenna by controlling the chemical potential of the graphene-based driven and parasitic elements. The antenna’s radiation direction is reconfigurable, covering a $360^{\circ }$ angle with four orthogonal beams ($0^{\circ }, 90^{\circ }, 180^{\circ }$ and $270^{\circ })$ at an operational frequency of 1.25 THz. Moreover, the antenna provides ultra wideband of bandwidth about $120\%$, the gain of 14 dBi and a front to back ratio of 15.5 dB.
- Furthermore, the experimental feasibility and the required bias voltage to enable the beam reconfiguration of the proposed antenna is discussed.

## Results

To evaluate the proposed graphene-based THz antenna structure, it is essential to model the conductivity of graphene at THz frequencies. According to Kubo formalism, the surface conductivity of graphene in the THz band can be approximated as^29^

1 $$\begin{aligned} \begin{aligned} \sigma _s&= -j\frac{e^2K_BT}{\pi \hbar ^2 (\omega -j\tau ^{-1})} \left[ \frac{\mu _c}{K_BT}+2\ln \left( \exp {\left( -\frac{\mu _c}{K_BT} \right) }+1\right) \right] , \end{aligned} \end{aligned}$$

where *j* is the imaginary unit, $K_B$ is the Boltzmann’s constant, *e* is the electronic charge, *T* is the temperature, $\omega$ is the angular frequency, $\hbar$ is the reduced Planck’s constant, $\tau$ is the relaxation time and $\mu _c$ is the chemical potential.

The conductivity of graphene is strongly dependent on the chemical potential $\mu _c$ and can be tuned by controlling the chemical potential $\mu _c$ level. Specifically, the chemical potential $\mu _c$ can be dynamically adjusted using electric field effect by means of chemical doping or DC bias voltage, thereby tuning the graphene conductivity. An applied electric field bias injects more electron or hole carriers, which allows to dynamically control the complex conductivity. The applied electric field *E* can be approximated as^30^.

2 $$\begin{aligned} \begin{aligned} E = \frac{q_e}{\pi \epsilon _0\hbar ^2v_f^2 } \int _{0}^{\infty }\varepsilon \left[ f_d(\varepsilon )- f_d(\varepsilon + 2 \mu _c )\right] d\varepsilon , \end{aligned} \end{aligned}$$

where $f_d(\varepsilon )$ = $[e^{(\varepsilon -\mu _c)/k_BT} +1]^{-1}$ is the Fermi-Dirac distribution.

Moreover, the chemical potential parameter of graphene can be adjusted by applying a bias voltage to the graphene layer, which can be approximated as^31^

3 $$\begin{aligned} \mu _c= {\hbar v_f}\sqrt{\frac{\pi C V_g}{e}}, \end{aligned}$$

where $C =\varepsilon _r\varepsilon _0 /t$ is the electrostatic capacitance, $\varepsilon _r$ is the relative permittivity of the substrate, $\varepsilon _0$ is the absolute permittivity in free space, *t* is the thickness of the substrate, and $v_f$ is the Fermi velocity of the graphene.

The bias voltage $V_g$ can be approximated from (3) as

4 $$\begin{aligned} V_g=\frac{e\mu _c^{2}t }{\pi \hbar ^2 v_f^{2}\varepsilon _0\varepsilon _r }. \end{aligned}$$

In the design of the proposed antenna, the chemical potential $\mu _c$ of 0.4 eV, 0.6 eV and 0.8 eV are considered to achieve the director, driven and reflector state. The changing of chemical potential parameter enables the reconfiguration in the graphene THz antenna. Experimentally, the variation of chemical potential is achieved by applying different bias voltages to graphene strips^32^. For instance, in the present case, the chemical potential $\mu _c$ = 0.4 eV is achieved with a bias voltage $V_b$ = 1.81 V, generating a static electric field *E* = 9.05 kV/cm. With the increase in the applied bias voltage, more charge is induced on the graphene element, which in turn increases the chemical potential. The tunable chemical potential of graphene enables the different states and reconfigurations in the graphene-based THz antennas. In the present work, the tunable conductivity of graphene via chemical potential is used to reconfigure the main beam into different directions.

**Figure 1:**
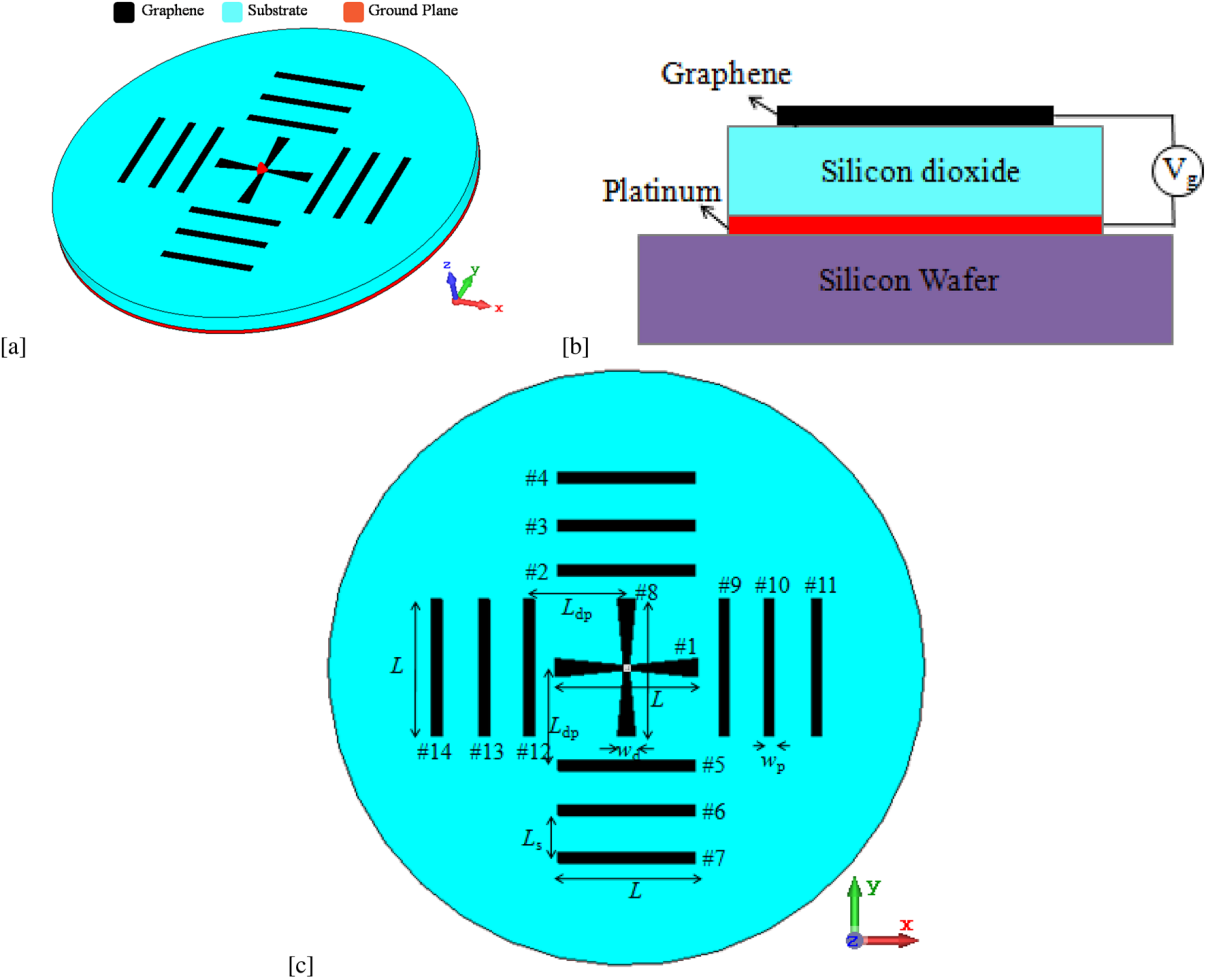
Schematic of the proposed graphene-based THz antenna. (**a**) 3D view (**b**) Cross-sectional view, and (**c**) Top view.

A graphene-based Yagi-Uda reconfigurable antenna with four orthogonal beams at THz band is investigated. The design of the proposed antennas is based on the principles of the Yagi-Uda antenna. The three-dimensional (3D) view, cross-sectional view, and top view of the proposed graphene-based Yagi-Uda antenna are shown in Fig. 1a, b, and c, respectively. The antenna consists of a centre-fed graphene-based driven dipole and three graphene-based parasitic elements on either side of the driven element. These graphene-based parasitic elements act as reflectors or directors depending on the chemical potential of the graphene. All graphene antenna elements are placed over a metallic grounded silica ($\text{SiO}_{2}$) substrate of circular geometry. The radius and height of the substrate are 100 µm and 2 µm, respectively. The platinum metal is used as a ground plane for the graphene THz antenna structure. The dimensions of the antenna are optimized for 1.25 THz operational frequency. For this frequency of the antenna, the values of geometrical dimensions of antenna elements are given in Table 1. Due to the propagation of SPP waves in graphene at THz, the proposed graphene THz antenna resonates at a sub-wavelength scale. The SPP wavelength is approximately 2.5 less than the free space wavelength ($\lambda _{SPP} \approx \lambda _0/2.5$) at operating frequency 1.25 THz. The driven element length of 48 µm (= $\lambda _{SPP}/2$) and spacing of reflector/director elements of 15 µm ($\approx 0.6 \lambda _{SPP}$) are considered in this work.

**Table 1:** Geometric parameters of the proposed antenna.

Parameters	*L* (µm)	$L_{dp}$ (µm)	$L_s$ (µm)	$W_d$ (µm)	$W_p$ (µm)
Values	48	28	15	6	3.5

In the proposed graphene-based Yagi-Uda THz antenna, the graphene-based parasitic elements either act as directors or reflectors by controlling the surface conductivity of these elements. The surface conductivity of the elements is adjusted individually by applying a bias voltage via the chemical potential of the graphene. The chemical potential of the graphene strip determines its conductivity and hence its active charge concentration region or electrical length. The reflectors are the graphene parasitic elements with higher chemical potential of graphene strip, which means a larger electrical length. The directors are the graphene parasitic elements with smaller chemical potential, which means a smaller electrical length. The dependency of graphene conductivity on the chemical potential parameter enables the reconfiguration in the graphene THz antenna. In the present work, the chemical potential $\mu _c$ values for the driven dipole, director and reflector are 0.6, 0.4 and 0.8 eV, respectively, which are listed in Table 2. If the chemical potential of graphene-based parasitic element is $\mu _c$ = 0, means no bias is applied to graphene-based element. The required active charge concentration region or electrical length to become reflector, director or driven dipole element is not fulfilled. Henceforth, the graphene based element with $\mu _c$ = 0 neither act as a reflector, director nor driven. Table 3 shows the dependence of chemical potential on bias voltage for the proposed antenna structure. From Table 3, it can be noticed that the bias voltage of 1.8 V, 2.7 V, and 3.6 V are required to enable the beam reconfiguration of the proposed antenna structure.

**Table 2:** Condition for different state elements.

Elements	Driven	Director	Reflector
$\mu _c$ values (eV)	0.6	0.4	0.8

**Table 3:** Bias voltage and corresponding chemical potential of graphene for the proposed antenna.

Bias voltage $V_b$ (V)	Electric field *E* (kV/cm)	Chemical potential $\mu _c$ (eV)
1.81	9.05	0.4
2.72	13.60	0.6
3.63	18.15	0.8

The proposed THz antenna consists of two graphene-based driven dipole elements (1 and 8) at the middle and 12 graphene-based parasitic elements on either side of the driven element. In addition, 2, 3, and 4 are three parasitic elements on top; 5, 6, and 7 are three parasitic elements on bottom; 9, 10, and 11 are three parasitic elements on left side; 12, 13, and 14 are three parasitic elements on right side. Feeding has been provided at the centre of driven elements 1 and 8. Two driven dipole elements 1 and 8 are placed in crossed manner at the middle, whereas as three parasitic elements are placed with equidistant in top, bottom, left and right side . The proposed graphene THz antenna operates in four different modes and provides four orthogonal beams. For each working mode, one driven element, one reflector and three directors are required. In mode *D*1, the element 1 is the driven dipole, elements 2, 3, and 4 are directors and the element 5 is the reflector. Similarly, the element 8 acts as the driven, the element 12 as the reflector and elements 9, 10, and 11 as the directors for mode *D*2; the element 1 as the driven, the element 2 as the reflector and elements 5, 6, and 7 as the directors for mode *D*3; the element 8 as the driven, the element 9 as reflector and elements 12, 13, and 14 as the directors for mode *D*4. The four modes with the chemical potential values of graphene antenna elements are listed in Table 4.

**Table 4:** Condition for different main beam direction.

Graphene element	Chemical potential of graphene element for different directions			
	$D_{1}$ (0°)	$D_{2}$ (90°)	$D_{3}$ (180°)	$D_{4}$ (270°)
1	0.6	0	0.6	0
2	0.4	0	0.8	0
3	0.4	0	0	0
4	0.4	0	0	0
5	0.8	0	0.4	0
6	0	0	0.4	0
7	0	0	0.4	0
8	0	0.6	0	0.6
9	0	0.4	0	0.8
10	0	0.4	0	0
11	0	0.4	0	0
12	0	0.8	0	0.4
13	0	0	0	0.4
14	0	0	0	0.4

**Figure 2:**
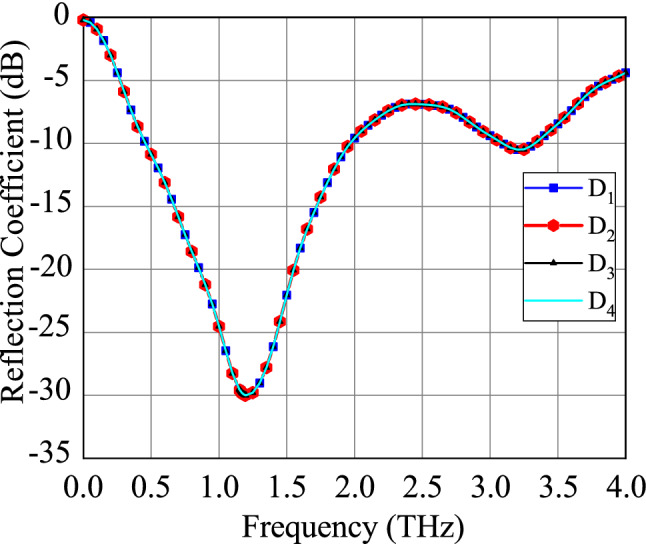
$S_{11}$ parameter of the proposed graphene THz antenna for four working modes.

**Figure 3:**
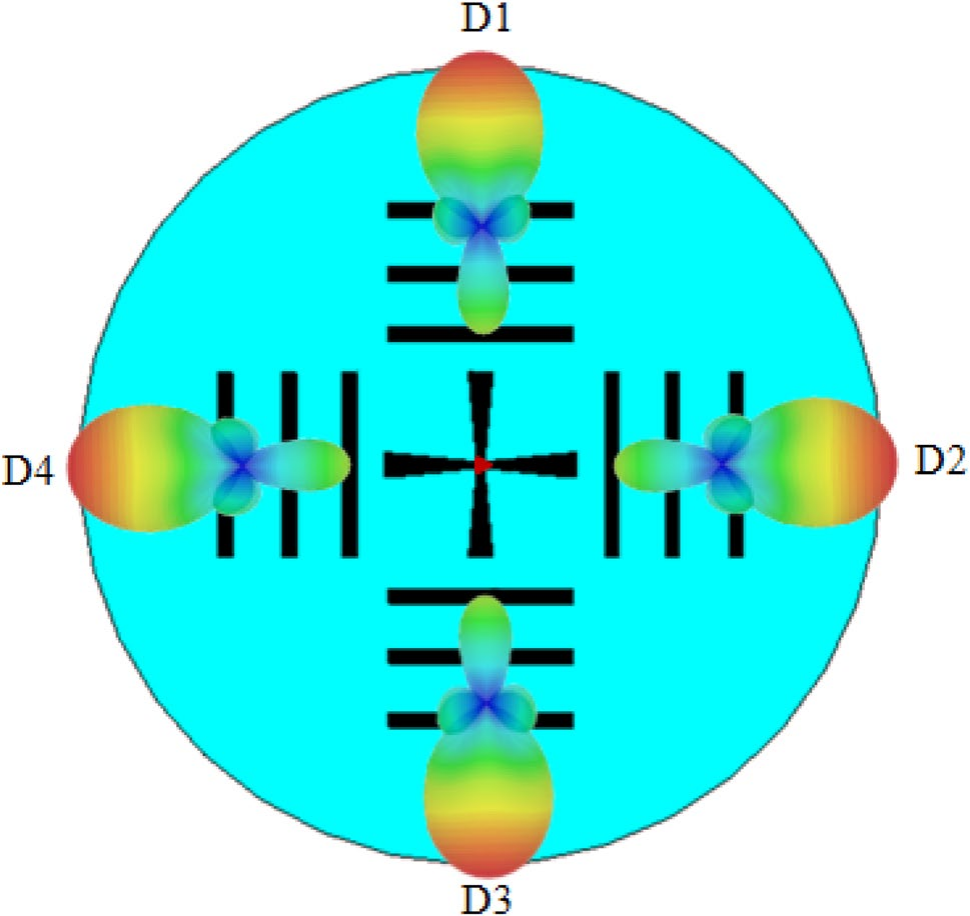
Four working modes of the graphene THz antenna.

**Figure 4:**
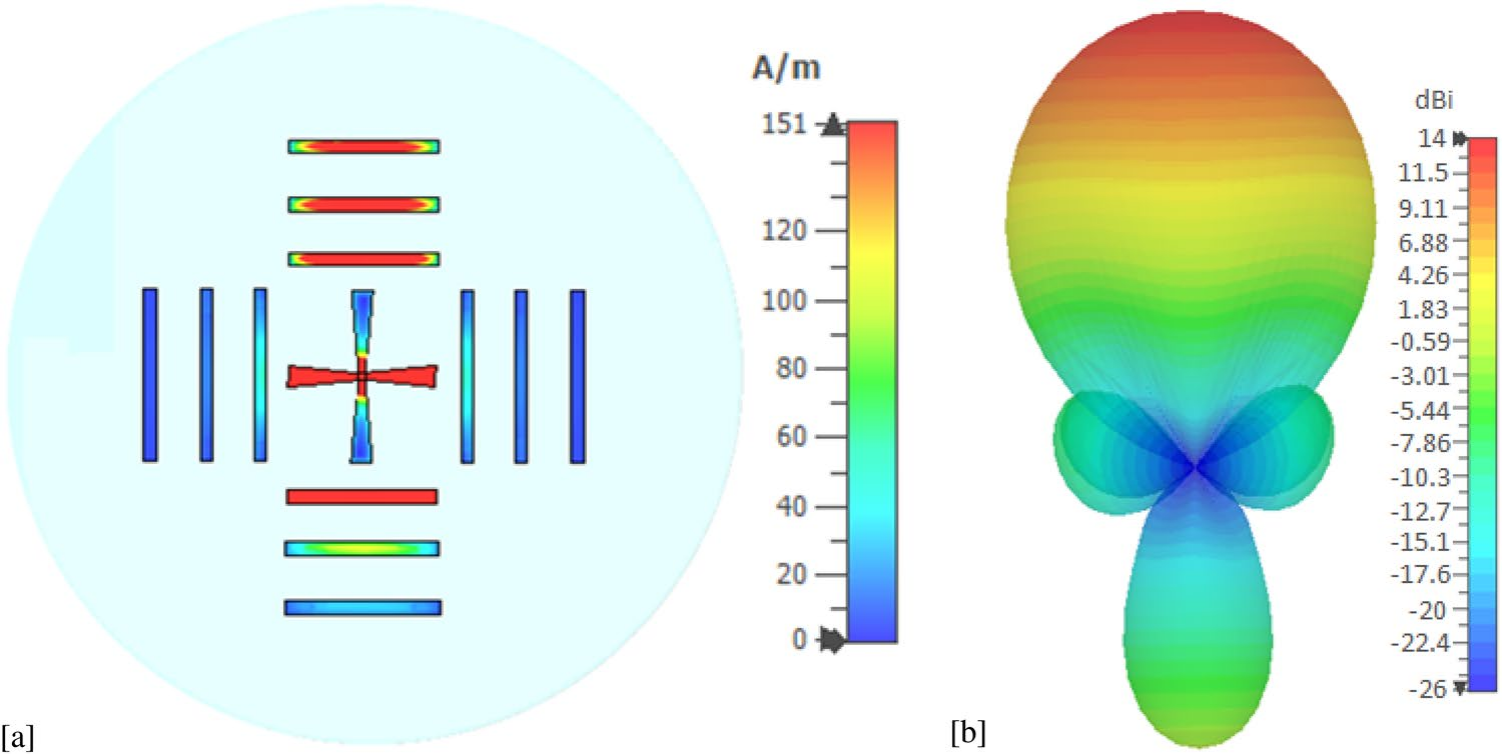
(**a**) Current distribution and (**b**) 3D far-field radiation pattern of the antenna at 1.25 THz in mode 1.

**Figure 5:**
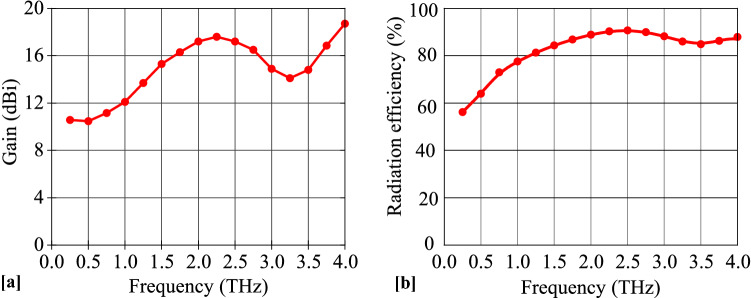
(**a**) Radiation Gain and (**b**) Radiation efficiency of the antenna over the frequency band $0-4$ THz.

The graphene properties and the dimensions are chosen for the antenna to operate at 1.25 THz frequency. The $S_{11}$ parameter of the antenna in four working modes $D_1, D_2, D_3$ and $D_4$ are shown in Fig. 2. Due to the plasmonic wave propagation in graphene at THz, the proposed graphene THz antenna resonates at a sub-wavelength scale. At 1.25 THz frequency, the SPP wavelength $\lambda _{SPP}=\lambda _{0}/2.5$, where $\lambda _{0}$ is the wavelength of free space. Moreover, the antenna provides an ultra wideband of 10-dB impedance bandwidth of $120\%$. The tunable graphene conductivity makes it possible to allow four different operation states. Using this unique tunable property, the reconfigurable antenna with four directional beams is presented in Fig. 3. The surface current distributions and the 3D far-field radiation pattern of the proposed antenna in mode $D_1$ are presented in Fig. 4. The antenna attains a unidirectional symmetrical radiation pattern with a gain of 14 dBi. The gain and radiation efficiency of the proposed graphene-based Yagi-Uda THz antenna over the frequency band 0–4 THz are shown in Fig. 5. It can be seen that the gain of the antenna is above 10 dBi over the frequency band 0–4 THz and the radiation efficiency of the antenna is above 80% over the frequency band 0–4 THz.

**Figure 6:**
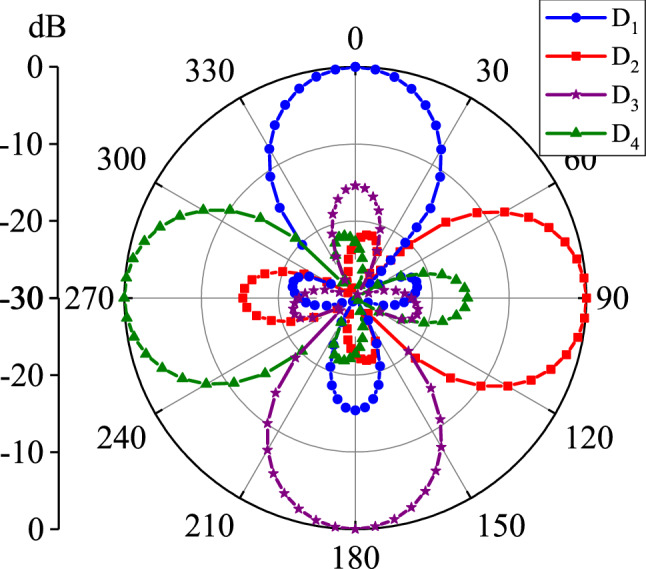
The normalized radiation pattern of the graphene THz antenna in four working modes.

**Figure 7:**
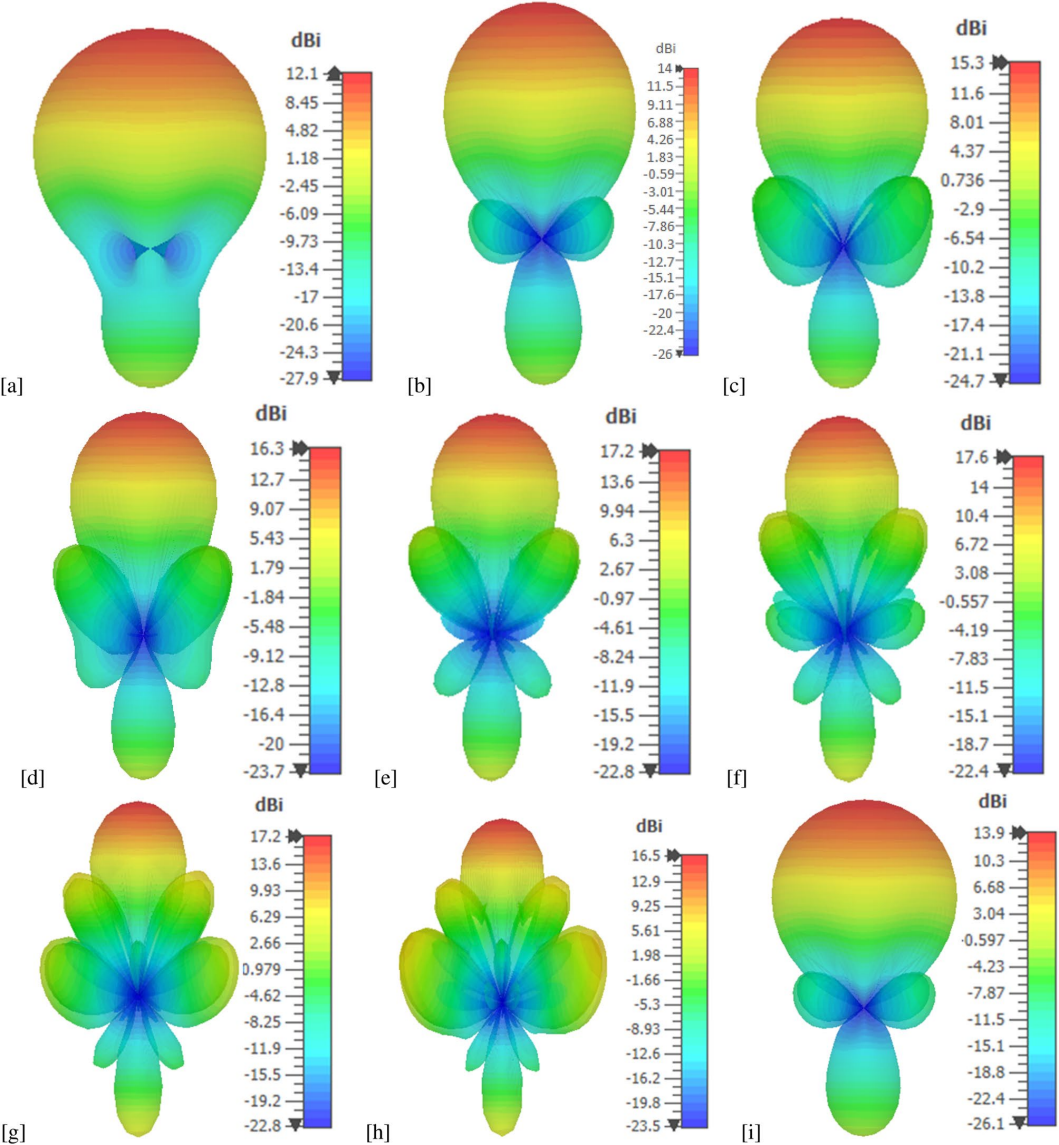
3D far-field radiation pattern of the antenna at (**a**) 1 THz, (**b**) 1.25 THz, (**c**) 1.5 THz, (**d**) 1.75 THz, (**e**) 2 THz, (**f**) 2.25 THz (**g**) 2.5 THz, (**h**) 2.75 THz, and (**i**) 3 THz in mode 1.

The normalized radiation patterns of the antenna in four working mode is shown in Fig. 6. By appropriately selecting the driven, director and reflector elements, the antenna can be directed in $D_1 (\theta =0^{\circ }), D_2 (\theta =90^{\circ }), D_3 (\theta =180^{\circ })$, and $D_4 (\theta =270^{\circ })$ directions. The antenna has the capability to reconfigure its radiation direction, covering a 360$^{\circ }$ angle at operational frequency 1.25 THz. Figure 7 presents 3D far-field radiation patterns of the proposed antenna in different frequency points ranging from 1 to 3 THz. Similar to the classical Yagi-Uda structure, the present proposed antenna employs a single reflector and more than one director. In the present work, a director and reflector are the graphene strip with chemical potential $\mu _c = 0.4$ eV and $\mu _c = 0.8$ eV, respectively. This is in contrast to a classical Yagi-Uda antenna structure, in which the parasitic elements get the induced current only from the excited element. Very few graphene-based Yagi-Uda THz antennas are available in the literature. We have compared the performance of the present antenna with graphene-based Yagi-Uda THz antennas reported in the literature, in Table 5. We can notice that the present antenna design has promising result in terms of antenna gain, band width, efficiency and front to back ratio.

**Table 5:** Performance comparison of proposed antenna with previously published graphene-based Yagi-Uda THz antenna.

Reference paper	^24^	^21^	^28^	^26^	^27^	This work
Radiator material	Metal (graphene as switches)	Graphene	Graphene	Graphene	Metal-graphene	Graphene
Operating frequency (THz)	1.88	1	2.5	1.243	2.97	1.25
Impedance bandwidth	10%	NR	12.38%	10.5%	5.04%	120%
Radiation efficiency	NR	NR	NR	NR	NR	82%
Gain /Directivity (dBi)	6.4	NR	9.78	6.5	3.7	14
Front-to-back ratio (dB)	12	NR	13.67	12.1	NR	15
Pattern reconfiguration	yes	yes	yes	yes	No	yes
Covering angle	0°–360°	− 42°–42°	− 75°–75°	0°–360°	− 70°–70°	0°–360°

*NR* Not reported.

## Discussion

The major concern in THz communications is the significant path loss effects on account of molecular absorption^33^. As a result, in order to achieve high data transfer rates, THz systems require devices with multiple antennas and beamforming capabilities. The proposed multibeam antennas are expected to meet this requirement. In traditional low-frequency smart antennas, complex signal processing is required to make a decision for the switched-beam antenna as to which beam to access at any time. In contrast, in the proposed THz antennas, the complexity is reduced significantly because only a voltage change is needed for steering the beam direction. The proposed THz antennas can act as promising candidate for THz communication systems due to its orthogonal beam patterns.

Recently, intelligent wireless environments have been proposed as promising smart environments that can make capable of sensing and manipulating wavefronts emitted by user devices^34,35^. This kind of environments are enabled by reconfigurable intelligent surfaces, a novel class of planar materials that combine the physics of meta-materials with the connectivity and deployment requirements of the Internet of Things. The unique plasmonic and electronic properties of graphene enable efficient reconfigurable intelligent surface at THz^36,37^. The user-side multibeam THz antennas and the environment-side intelligent surfaces can build a promising THz wireless system.

## Conclusion

A graphene-based wideband beam reconfigurable directional antenna for THz wireless communication system is proposed in this work. The concept is based on the working principle of the traditional Yagi-Uda antenna. The graphene-based parasitic elements either act as directors or reflectors by controlling the surface conductivity of these elements. The surface conductivity of the elements is adjusted individually by applying a bias voltage via the chemical potential of the graphene. The tunable conductivity behaviour of graphene is used to create orthogonal beams. The performance of the proposed antenna is controlled by the chemical potential of the graphene-based driven and parasitic elements. The antenna is reconfigured in its radiation direction, covering a 360$^{\circ }$ angle with four orthogonal beams ($0^{\circ }, 90^{\circ }, 180^{\circ }$ and $270^{\circ }$) at the operational frequency 1.25 THz. Moreover, the antenna provides ultra wideband of bandwidth about $120\%$, the gain of 14 dBi and the front to back ratio of 15.5 dB. The results reveal that the proposed graphene-based THz antenna is promising for THz wireless communication systems. As the future wireless communication system will rely on THz frequency in a big way, the proposed ultra-wideband high gain beam reconfigurable THz antenna structure will cater to the need of the ever-growing wireless communication users.

## Method

The CST Studio Suite EM simulator is used to model the graphene and validate the proposed designed antenna^38^. In the EM simulator, the graphene element is modelled as a thin resistive sheet with surface impedance $Z_s={1}/{\sigma _s}$, where $\sigma _s$ is the surface conductivity of graphene and depends on angular frequency $\omega$, chemical potential $\mu _c$, relaxation time $\tau$ and temperature *T*. The thickness of graphene strip $t_g = 1$ nm is considered^39^. In the present work, room temperature *T* = 300 *K*, Fermi velocity $v_f = 10^6$ m/s and mobility of graphene $\mu _{g} = 10000$ $\textrm{cm}^2/\textrm{Vs}$ are considered^40^. The relaxation time of graphene is computed as $\tau$ = ${\mu _{g} \mu _{c} }/{ev_{f}^{2}}$^3^. The discrete face port (fed by a 50 ohm source) is considered for the excitation of the antenna structure in the CST Studio Suite EM simulator. For mode $D_1 (\theta =0^{\circ })$ and $D_3 (\theta =180^{\circ })$, excitation is provided at the centre of driven element 1, whereas for mode $D_2 (\theta =90^{\circ })$ and $D_4 (\theta =270^{\circ })$, excitation is provided at the centre of driven element 8. The performance of the proposed graphene THz antenna model is studied in the wide spectrum from 0 to 4 THz.

In the EM simulator, the changing of chemical potential parameter enables the reconfiguration in the graphene THz antenna. To achieve the different modes in the proposed graphene THz antenna, the chemical potential of graphene elements varies from 0.4 eV to 0.8 eV. The variation of chemical potential is achieved by applying different bias voltages to graphene strips. In the present case, the chemical potential $\mu _c$ = 0.4 eV is achieved with a bias voltage $V_b$ = 1. 81 V, generating a static electric field *E* = 9.05 kV/cm. With the increase in the applied bias voltage, more charge is induced on the graphene strip, which in turn increases the chemical potential. The bias voltage of 1.8, 2.7, and 3.6 V are required to enable the beam reconfiguration of the proposed antenna structure.

The feasibility of the fabrication of the proposed antenna structure can be explained with the help of Fig. 1b. Mechanical support of graphene-based Yagi-Uda antenna structure can be provided by using a silicon wafer sample. Using the e-gun evaporation technique, a thin polysilicon layer of 100 nm can be evaporated on the silicon wafer sample surface^41^. On top of the polysilicon layer, a silicon dioxide layer of thickness 2 µm can be spin-coated and then, it should be heated at 300 °C for one hour. The chemical vapour deposition grown graphene strips can then be transferred onto the silicon dioxide layer. Graphene conductive ink is also promising for printed electronics. The antenna can also be fabricated using graphene ink on the silicon dioxide substrate.

The reconfigurable capabilities of the proposed graphene THz antenna structure can be demonstrated experimentally by independent control of each graphene strip through different biasing gates^32,42,43^. The different bias voltages enable the different chemical potential on graphene, leads to control of surface conductivity in graphene sheets. The tunable conductivity nature of the graphene elements in the proposed antenna enables beam reconfigurability. The modulation of graphene conductivity experimentally can also be achieved by a photoexcitation process^41^.

## Data Availability

All data generated or analysed during this study are included in this manuscript.
